# Mindful Exercise (Baduanjin) as an Adjuvant Treatment for Older Adults (60 Years Old and Over) of Knee Osteoarthritis: A Randomized Controlled Trial

**DOI:** 10.1155/2020/9869161

**Published:** 2020-06-14

**Authors:** JiaJia Ye, Qikai Zheng, Liye Zou, Qian Yu, Nicola Veronese, Igor Grabovac, Sinisa Stefanac, Huey-Ming Tzeng, Jane Jie Yu

**Affiliations:** ^1^Department of Rehabilitation Assessment, Rehabilitation Hospital Affiliated to Fujian University of Traditional Chinese Medicine, Fuzhou, Fujian 350000, China; ^2^Fujian Key Laboratory of Rehabilitation Technology, 13 Hudong Road, Fuzhou, Fujian 350000, China; ^3^Department of Rehabilitation Sciences, Hong Kong Polytechnic University, Hong Kong, China; ^4^Department of Athletic Injury, Rehabilitation Hospital Affiliated to Fujian University of Traditional Chinese Medicine, Fuzhou, Fujian 350000, China; ^5^Exercise and Mental Health Laboratory, Shenzhen University, Shenzhen 518060, China; ^6^National Research Council, Neuroscience Institute, Aging Branch, Padua, Italy; ^7^Department of Social and Preventive Medicine, Centre for Public Health, Medical University of Vienna, Vienna, Austria; ^8^The University of Texas Medical Branch at Galveston, School of Nursing, 301 University Boulevard, Galveston, Galveston, TX 77555, USA; ^9^Department of Sports Science and Physical Education, The Chinese University of Hong Kong, Shatin, New Territories, Hong Kong 999077, China

## Abstract

**Background:**

The postural stability is a major factor that helps prevent developing knee osteoarthritis with aging. The aim of this study was to investigate the effects of Baduanjin qigong on postural control and physical function in older adults with knee osteoarthritis.

**Methods:**

Fifty-six individuals over 60 years of age with knee osteoarthritis were randomly assigned to either an experimental group (*n* = 28) or a control group (*n* = 28). Participants in the experimental group received a 12-week Baduanjin training, while those in the control group did not receive any additional physical exercise during the study period. The postural control was quantified by perimeter and ellipse area of center of pressure movement trajectory. The assessments were conducted three times (baseline, week 8, and week 12).

**Results:**

The perimeter and ellipse area with both open- and closed-eyes conditions and Western Ontario and McMaster Universities Osteoarthritis Index (WOMAC) function were significantly improved at week eight in the experimental group (*p* < 0.005). The ellipse area with open-eyes condition, WOMAC index, and stiffness and physical function domains were significantly decreased after the 12 weeks of Baduanjin training compared to the control group (*p* < 0.005). Only the perimeter area with both open- and closed-eyes conditions was not statistically significant at week 12 in the intervention group (*p* > 0.005).

**Conclusions:**

Baduanjin is an effective and adjuvant therapy for older adults with knee osteoarthritis. Regular Baduanjin training can improve postural control and WOMAC function of old individuals with knee osteoarthritis. More advanced techniques and biopsychological measurements are required for further understanding of Baduanjin exercise in this population. The trial was registered in Chinese Clinical Trial Registry (ChiCTR-IOR-16010042).

## 1. Introduction

Knee osteoarthritis (KOA) is one of the most commonly occurring degenerative joint illnesses among people over 60 years old, and it commonly causes pain, poor balance, disability, and increased risk of falling [1]. According to the National Health Interview Survey, there are approximately 14 million US citizens suffering from KOA [2]. Furthermore, data from the 2017 National Longitudinal Survey has indicated that nearly 8% of the older Chinese population were diagnosed with symptomatic KOA, with people living in rural areas of China being twice as likely to have increased risk of all-cause-mortality primarily caused by decreased postural stability or balance-associated outcomes [3]. Results from nationally representative studies indicated a substantial burden from KOA in Chinese older adults and authors have called for an urgent need for cost-effective intervention programs such as exercise, self-management, and active coping strategies [4].

Baduanjin (BD) is a mind-body exercise with mild-to-moderate intensity. Its entire choreographic form consists of eight movements performed at a slow pace in coordination with rhythmic breathing, mental focus (on muscle and movement sense), and meditative state of mind [5]. Such culture-specific exercise can spark the interest of Chinese older adults, especially this age group who suffer from chronic diseases [6, 7]. Indeed, as a part of traditional Chinese medicine, BD has been commonly used in hospital setting for treating Chinese people with psychiatric disorders, ankylosing spondylitis, type 2 diabetic mellitus, Parkinson's disease, fatigue syndrome-like illness, insomnia, and essential hypertension [7–10]. Furthermore, results from a recent systematic review of randomized controlled trials (RCT) showed the beneficial effects of BD-based interventions compared to controls in three domains of WOMAC (pain, stiffness, and physical function) [11].

So far, only one study has focused on the effects of BD intervention on postural stability showing that it deteriorated faster in KOA patients compared to older adults without KOA [12]. Postural control is a complex sensorimotor function with inputs of visual, vestibular, and somatosensory systems [13]. The interaction between these elements may generate a stabilization of posture that greatly reduces the incidence of falls [14]. Impaired postural control in older persons with KOA has been found associated with loss of quadriceps strength, proprioceptive deficit, and pain [14–17]. Furthermore, the underlying mechanisms regarding the postural instability of KOA may be attributed to the weak communication between sensory information and motor neuron, which can be moderated by center of pressure (COP) [18]. Given that movements in BD routine involve continuous weight-shifting and semisquatting position that coordinate with proprioceptive awareness on muscle and movement sense, it may provide the opportunity to train postural stability of KOA patients. In addition, as mind-body relaxation and breathing techniques are developed throughout a BD intervention, disease-specific symptoms (pain and stiffness) are possibly alleviated. Against this background, the aim of this study was to explore if postural stability and disease-related symptoms of KOA patients could be improved following a 12-week BD intervention using a RCT.

## 2. Materials and Methods

### 2.1. Study Design

This study was a pilot RCT investigating the effects of BD on postural stability among KOA patients over 60 years old. The procedure and results of this study were in line with the Consolidated Standards of Reporting Trials (CONSORT) guidelines [19]. All eligible KOA patients were randomly divided with a ratio of 1 : 1 to either an intervention group or control group using the statistical software SAS 9.1 by a clinical doctor. The numbers generated by computers were provided in sealed, opaque envelopes and opened on the participants' agreement to participate. Notably, both the intervention and control groups received the conventional therapies (acupuncture, massage, and moxibustion), one hour each day, five days per week in the first four weeks. Postural stability (primary outcomes) and three domains of WOAMC (secondary outcomes) were measured at baseline, week eight, and week 12. The clinical doctor was blind to the content of this study. All participants had provided written informed consent forms before their participation, which was carried out in accordance with the declaration of Helsinki. This study was reviewed and approved by the Medical Ethics Committee of the Affiliated Rehabilitation Hospital of Fujian University of Traditional Chinese Medicine (approval number: 2014KY-020-01). The patients/participants provided their written informed consent to participate in this study.

### 2.2. Study Participants

A number of 105 participants were recruited through referral from their doctors of the Rehabilitation Hospital between January 2015 and January 2016. Participants were considered eligible if they met the following criteria: (1) males and females aged over 60 years; (2) diagnosed with KOA according to criteria of the American College of Rheumatology [20], with a radiographic grading of the severity between 2 and 3; (3) had no training experience in any kinds of mind-body exercise (Tai Chi, Qigong, and/or Yoga) prior to six months before enrollment; (4) able to ambulate without any device assistance; (5) enrolled as an inpatient. The exclusion criteria were as follows: (1) participants with severe cardiovascular, respiratory, or other musculoskeletal diseases; (2) participants on medications that could affect the musculoskeletal system or postural stability (e.g., antidepressants, dopaminergic agents, and hypnotics); (3) participants who had a bone fracture within one year [21].

### 2.3. Intervention Program and Control Group

Patients in the intervention group received a 12-week BD program. Patients were asked to perform three BD sessions per week, with each session lasting for 40 minutes. This training scheme was consistent with a guideline recommended by the Chinese Health-Qigong Association [22]. The content of BD includes eight sections, namely, section 1: elevate both hands to the sky; section 2: draw a bow on both sides; section 3: raise single arm each time; section 4: look back; section 5: sway the head and shake the tail; section 6: touch toes by hands with flexion of hip and extension of knee joint; section 7: clench fists; section 8: bounce on the toes. Patients in the intervention group took part in group-based BD training sessions in the hospital for the first four weeks under the supervision of a qualified instructor. After the initial in-hospital training, participants were instructed to continue to practice at home for the remaining time (till week 12). In order to maximize adherence, all participants were asked to keep a daily log and the research team checked in via telephone. Participants in the control group were asked to maintain their normal lifestyle and received the same BD training information after the study end.

### 2.4. Outcome Measures

The demographic and anthropometric data were collected at baseline. The assessments of perimeter and ellipse area of center of pressure (COP) and self-administered measurement (WOAMC) were taken at three time points: baseline (week 0), week eight, and week 12. Research associates were blinded to the group allocation during assessments. All eligible participants were informed to come to the rehabilitation laboratory one week before the actual assessment to familiarize with measurements.

#### 2.4.1. Primary Outcome Measures

**Figure 1:**
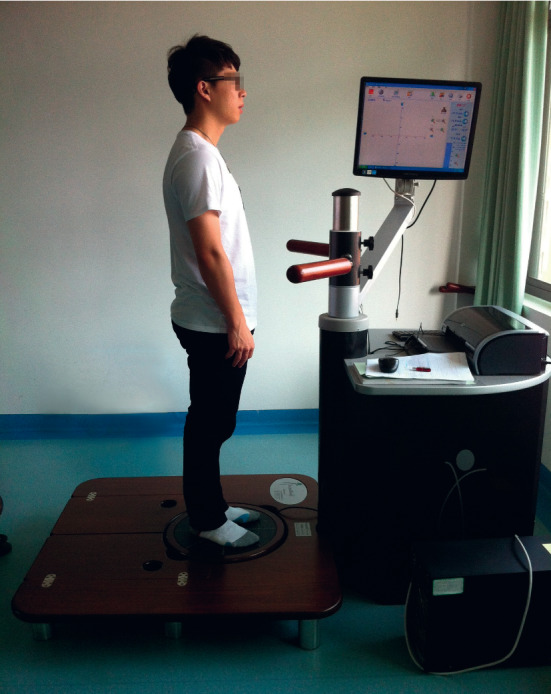
Postural control measurement on ProKin system.

The length and the area of COP movement trajectory were measured using the ProKin system (PK-252, TecnoBody, Italy). All participants were instructed to stand on the force platform with barefoot bipedal and arms at their sides (Figure 1). Participants were tested in open- and closed-eyes conditions, with each lasting 30 seconds. The open-eyes test was first performed, followed by a closed-eyes test. In the first condition, participants were asked to focus on a fixation point on a wall 1.5 meters in front. Variations in COP were quantified by the perimeter in millimeters (mm) and ellipse area in square millimeters (mm^2^). The higher value of perimeter and ellipse area of COP indicated the worse ability of postural control [23].

#### 2.4.2. Secondary Outcome Measures

The Western Ontario and McMaster Universities Osteoarthritis Index (WOMAC) is widely used in the evaluation of pain, stiffness, and physical function in KOA patients with its high reliability and validity [24]. This is a self-administered assessment tool that involves 24 items with five items for pain, two items for stiffness, and 17 items for physical function. Eligible participants were informed to complete this measurement using a five-point Likert-scale within 15 minutes. Zero represents absence of pain, stiffness or impairment, and four represents extreme pain, stiffness, and difficulty.

### 2.5. Safety Record

A questionnaire probing possible adverse events and changes in health status was given to eligible participants. Adherence and occurrence of adverse events during the study were recorded on an adverse event case report form and were evaluated for relevance to the intervention by research team.

### 2.6. Data Analysis

The demographics and characteristics of participants were presented with descriptive statistics. Shapiro–Wilk test was performed to assess data normality. Baseline characteristics between groups were compared by the independent *t*-test. Two-way repeated measures analysis of variance (RM ANOVA) (group × time) was conducted to detect the interaction effect between group and time. If a significant interaction emerged, we used the simple effect tests. Post hoc pairwise comparisons with Bonferroni adjustment were used for following significant main effect. Independent *t*-test was conducted to compare the between-group differences in primary and secondary outcomes. All the statistical tests were performed by statistical software SPSS 20 (IBM, New York, USA), and *p* ≤ 0.05 was used to denote the statistical significance.

## 3. Results

**Figure 2:**
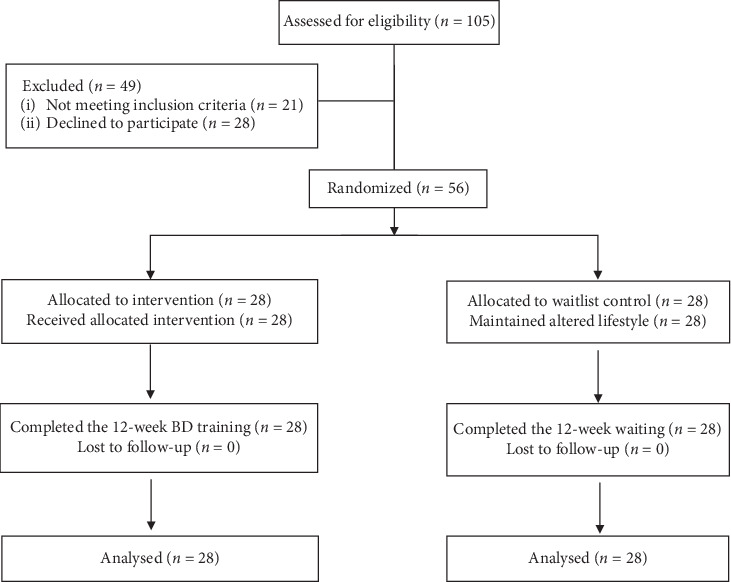
The detailed procedure of this study.

The demographic and anthropometric data of participants are shown in Table 1. A total of 105 patients were initially referred by their doctors, 49 of which were screened against the selection criteria. Fifty-six individuals were finally included and randomly assigned to the experimental group (*n* = 28, male = 11, female = 17) or the control group (*n* = 28, male = 8, female = 20). The average age was 65.11 ± 6.57 for BD and 63.61 ± 2.63 for control group. The flow chart of this RCT is presented in Figure 2.

### 3.1. Postural Stability

Significant condition-by-time interaction effects were observed on the ellipse area with open-eyes (*F* (1, 54) = 19.730, *p* = 0.001, *η*^2^ = 0.268) and closed-eyes conditions (*F* (1, 54) = 17.879, *p* = 0.001, *η*^2^ = 0.249) and the perimeter area with closed-eyes condition (*F* (1, 54) = 5.933, *p* = 0.005, *η*^2^ = 0.100) (Table 2). Furthermore, results from simple effect test showed that the BD group had significant improvements on the ellipse area with open-eyes (*p* = 0.001) and closed-eyes conditions (*p* = 0.001) at week 12 and the perimeter area with open-eyes condition (*p* = 0.018) compared to baseline (Table 3). Additionally, only BD group showed significant improvement of ellipse area with open-eyes condition at week 12 (MD = −235.64, *p* = 0.007) as compared to the control group (Table 2).

### 3.2. WOMAC

Significant group × time interaction effects were found in WOMAC (*F* (1, 54) = 15.922, *p*=0.001, *η*^2^ = 0.228), pain (*F* (1, 54) = 4.957, *p*=0.015, *η*^2^ = 0.084), and physical function (*F* (1, 54) = 14.302, *p*=0.001, *η*^2^ = 0.209), but not in stiffness (*F* (1, 54) = 0.927, *p*=0.399, *η*^2^ = 0.017) (Table 2). Results from the simple effect test indicate that significant improvements at week eight (WOMAC total scores, *p*=0.001; pain, *p*=0.001; stiffness, *p*=0.001; physical function, *p*=0.03) and week 12 (WOMAC total score, *p*=0.001; stiffness, *p*=0.001; physical function, *p*=0.001) were observed, whereas perceived stiffness was significantly reduced at week eight (*p*=0.001) and week 12 (*p*=0.001) in the control group compared to baseline (Table 3). Additionally, the BD group showed significantly greater improvements at week eight (WOMAC total score, MD = −9.0, *p*=0.003; perceived stiffness, MD = −4.43, *p*=0.001) and week 12 (WOMAC total score, MD = −12.97, *p*=0.001; perceived stiffness, MD = −3.83, *p*=0.001; physical function, MD = −6.04, *p*=0.001) compared to the control group (Table 2).

### 3.3. Safety Report

No adverse event occurred in both groups during the 12-week intervention period.

## 4. Discussion

The present study investigated the effects of a 12-week BD program on postural stability and disease-specific symptoms in patients with KOA who are over 60 years old. We found that regular BD training improved parameters (perimeter and/or ellipse area with open- and closed-eyes conditions) of postural stability, which is supported by a randomized controlled trial, suggesting that BD training effectively improved postural stability at the anterior–posterior direction with closed-eyes condition among KOA patients aged between 50 and 80 years [12]. In addition, these findings are in accordance with a recently published meta-analysis suggesting that, compared with waitlist control, BD training has favorable effects on pain (MD = −4.40, 95% CI: −7.16, −1.64, *p* < 0.01), stiffness (MD = −1.34, 95% CI: −1.64, −1.04, *p* < 0.01), and physical function (MD = −2.44, 95% CI: −4.33, −0.55, *p* < 0.01), related to control groups [11].

In the BD group, we found only a marginally significant reduction on ellipse area (small area indicates better postural stability) with closed-eyes condition (*p* = 0.064) at week eight. Such result seems to indicate that eight-week BD may be a minimal effective dosage to trigger a positive effect on the selected parameter of postural stability. However, a nonsignificant result on this parameter may be attributed to the fact that, in the first eight weeks, participants were more likely to concentrate on motor skill learning or master basic movements that involve more cognitive challenge (memory, visual-spatial ability, and executive function) but paid less attention to the quality of movement (e.g., footwork) in which participants were asked to maintain their balance in motion. Considering the fact that people aged 60 years experienced a cognitive decline, future studies should also include cognitive outcome measures of KOA patients before and after the BD intervention.

Interestingly, after the 12-week intervention period, the ellipse area with both open- and closed-eyes conditions was significantly reduced in both BD and control groups, but these results were not observed on the perimeter area. Ellipse area seems to be a more sensitive parameter that reflects postural stability compared to perimeter, and this requires further investigation. Furthermore, the BD group showed greater reduction in the ellipse area with open-eyes condition at week 12 compared with the control group, suggesting that BD as an adjunctive intervention in coordination with usual care has a more favorable effect on postural stability. Such result was only observed in KOA patients with open-eyes condition that may be attributed to improved visual-spatial ability following the 12-week BD program, compared with the control group who had not received such unique training throughout this intervention period [25]. This result may also be due to the fact that BD trainees effectively reduced level of pain-catastrophizing and improved self-efficacy that may mediate the effects of this mindful exercise (like Tai Chi) on postural stability [26, 27]. Collectively, protective effects of BD training on postural stability may be partially explained by the feature of dynamic movements. In performing BD form, practitioners were asked to control over their balance (center of gravity) while smooth moves are carried out with the lumbar spine as an axis [28]. Specifically, weight-shifting at four directions, especially at the upper body, constantly challenges postural stability of the lower limbs. For example, when performing movement five, people are required to circle their lumbar spine with approximately 45 degrees, while movement six requires a 90-degree forward-leaning of the upper body. In performing these unique movements, BD trainees were trained to maintain the center of gravity in their lower limbs, especially feet that should be rooted over the ground, without crossing the borderline of a shoulder width (similar to ellipse area).

With regard to symptoms of KOA patients in the BD group, we observed significant improvements on WOMAC total scores, pain, stiffness, and physical function at week 8, and these positive effects continued to appear until week 12. It may indicate that a minimal effective dosage was eight weeks (three sessions per week, lasting 40 min for each section). Notably, significant results in the control group was only observed on stiffness at both week eight and week 12, which may be attributed to acupuncture, moxibustion, and massage, as the commonly conventional therapies, received by the control group for ethical reasons. Previous studies indicated significant improvement on perceived stiffness was observed following an eight-week self-massage two times × 20 minutes per week [26]. Compared with the control group, BD group showed better performances in terms of selected measures of symptoms at week eight and week 12, suggesting that BD integrated with the conventional therapy was more effective for treating this symptom of KOA patients. Indeed, all movements in BD form involve musculoskeletal stretching, which reasonably contributes to an increased range of motion that is associated with a decrease of perceived stiffness. Collectively, these symptomatic measures were observed at a significant level since week eight. It may be associated with key components (meditative state of mind, mental focus, and relaxation) of BD as a traditional Chinese mindful exercise. These combined elements in mindful exercise (Tai Chi, Yoga, and mindfulness) or alone have been shown to be associated with a lower level of pain and better physical function [29, 30]. Once all individual subdomains improved, WOMAC total score is reasonably better.

### 4.1. Limitations and Future Research Directions

There are some limitations in this study. First, we only recruited participants in one hospital; findings from this exploratory study may not be extrapolated to other medical settings. Second, we did not investigate the relationship between postural control and each movement section. Third, we did not include any psychological measurement tools in current study. As the closed link between chronic pain, disability, and mental disorders has been found, future studies should include some mental measures (e.g. self-efficacy, depression, and anxiety). Fourth, results in the present study have indicated ellipse area seems to be more sensitive relative to perimeter area, which can be warranted by future studies. Fifth, some variables associated with postural stability were not measured in this study, including muscular strength, flexibility of lower limbs, and trunk muscles. Last, while low-limb proprioception, visual-spatial ability, and negative cognitive and emotional responses (like pain-catastrophizing) can be improved following Tai Chi. It is necessary to further investigate whether these variables can mediate the effects of BD on postural stability of KOA patients aged 60 years and over.

## 5. Conclusions

In conclusion, the current study concluded that the postural control and WOMAC function in older persons with KOA might be initially improved by eight-week BD training, and this positive effect could be continually found until week 12. It is suggested that a 12-week combined program BD training (hospital and home settings) with three sessions per week, lasting 40 min for each session, may have considerably beneficial effects on postural stability and WOMAC function in older adults with KOA, and BD is an adjunctive intervention in coordination with usual care that have a more considerable effect on postural stability. Additionally, the ellipse area is likely to be more sensitive in terms of reflecting a postural stability than the perimeter area.

## Figures and Tables

**Table 1:** Baseline characteristics of participants.

Characteristics	BD (*n* = 28)	CG (*n* = 28)	*p*
Gender (*n*)			
Male	11	8	
Female	17	20	0.678
Age (year)	65.11 ± 6.57	63.61 ± 2.63	0.237
Height (cm)	164.04 ± 7.73	163.32 ± 8.27	0.740
Weight (kg)	65.32 ± 8.97	65.61 ± 7.09	0.895
Body mass index (kg/m^2^)	24.19 ± 2.37	24.63 ± 2.27	0.439

BD, Baduanjin group; CG, control group.

**Table 2:** Comparison of postural stability and WOMAC function between the BD and control groups over time.

Variable	BD	CG	MD	*p* value (95% CI)	Time effect	Group × time effect		
					*p*	*p*	*F*	*η * ^2^
OP perimeter (mm)					0.176	0.180	1.756	0.031
Baseline	379.86 ± 272.80	364.32 ± 190.45	15.54	0.806 (−110.52∼141.59)				
Week 8	367.07 ± 199.36	381.50 ± 196.21	−14.43	0.786 (−120.14∼91.55)				
Week 12	252.64 ± 129.22	346.71 ± 393.78	−94.07	0.235 (251.10∼62.96)				
OP ellipse area (mm^2^)					0.001^*∗*^	0.001^*∗*^	19.730	0.268
Baseline	799.96 ± 334.53	860.07 ± 223.79	−60.11	0.433 (−212.60∼92.39)				
Week 8	704.46 ± 295.06	774.36 ± 134.91	−69.90	0.259 (−192.82∼53.03)				
Week 12	391.93 ± 260.39	627.57 ± 358.06	−235.64	0.007^*∗*^ (−403.39∼−67.90)				
CP perimeter (mm)					0.003^*∗*^	0.005^*∗*^	5.993	
Baseline	532.39 ± 2252.33	509.36 ± 192.20	23.03	0.702 (−97.14∼143.22)				
Week 8	553.50 ± 201.31	508.86 ± 243.19	44.64	0.458 (−74.97∼164.26)				
Week 12	450.14 ± 215.22	346.71 ± 393.78	103.43	0.564 (−76.71∼139.28)				
CP ellipse area (mm^2^)					0.001^*∗*^	0.001^*∗*^	17.879	0.249
Baseline	1248.11 ± 389.57	1147.29 ± 555.52	100.82	0.435 (−156.25∼357.90)				
Week 8	1057.43 ± 384.26	1026.14 ± 322.56	31.29	0.743 (−158.80∼221.37)				
Week 12	694.61 ± 305.31	820.93 ± 419.04	−126.32	0.203 (−322.76∼70.12)				
WOMAC					0.001^*∗*^	0.001^*∗*^	15.922	0.228
Baseline	30.57 ± 11.88	28.21 ± 11.18	2.36	0.448 (−3.82∼8.54)				
Week 8	19.39 ± 9.60	28.39 ± 11.57	−9.00	0.003^*∗*^(−14.70∼−3.30)				
Week 12	15.32 ± 6.56	28.29 ± 7.96	−12.97	0.001^*∗*^ (−16.87∼−9.06)				
Pain					0.009^*∗*^	0.015^*∗*^	4.957	0.084
Baseline	6.21 ± 2.67	7.36 ± 8.89	−1.15	0.517 (−4.66∼2.37)				
Week 8	3.04 ± 1.53	6.21 ± 2.41	−3.17	0.001^*∗*^ (−4.26∼−2.10)				
Week 12	3.79 ± 5.83	4.64 ± 1.91	−0.85	0.463 (−3.18∼1.47)				
Stiffness					0.531	0.399	0.927	0.017
Baseline	4.04 ± 2.05	4.25 ± 1.38	−0.21	0.648 (−1.15∼0.72)				
Week 8	2.36 ± 1.64	6.79 ± 2.56	−4.43	0.001^*∗*^ (−5.58∼−3.28)				
Week 12	2.46 ± 1.58	6.29 ± 2.11	−3.83	0.001^*∗*^ (−4.82∼−2.83)				
Physical function					0.001^*∗*^	0.001^*∗*^	14.302	0.209
Baseline	20.36 ± 10.52	18.25 ± 8.04	2.11	0.409 (−2.98∼7.19)				
Week 8	14.43 ± 9.10	15.68 ± 9.09	−1.25	0.609 (−6.122∼3.622)				
Week 12	9.96 ± 5.95	16.00 ± 6.54	−6.04	0.001^*∗*^ (−9.38∼−2.69)				

OP, open-eyes; CP, closed-eyes; BD, Baduanjin group; CG, control group; WOMAC, the Western Ontario and Mcmaster Universities Osteoarthritis Index; MD, mean change. ^*∗*^Significant effect (*p* < 0.05).

**Table 3:** Comparison of postural stability and WOMAC function over time in both groups.

Variable		Week 0 vs. week 8	Week 8 vs. week 12	Week 0 vs. week 12
BD				
OP	*p*	0.828	0.013^*∗*^	0.018^*∗*^
Perimeter (mm)	95% CI	−106.86 to 132.43	26.56 to 202.29	23.13 to 231.31
OP	*p*	0.226	0.001^*∗*^	0.001^*∗*^
Ellipse area (mm^2^)	95% CI	−62.78 to 253.78	161.29 to 463.78	223.94 to 592.13
CP	*p*	0.693	0.001^*∗*^	0.052
Perimeter (mm)	95% CI	−129.46 to 87.24	26.87 to 179.85	−0.67 to 165.17
CP	*p*	0.064	0.001^*∗*^	0.001^*∗*^
Ellipse area (mm^2^)	95% CI	−12.05 to 393.41	474.27 to 856.73	391.38 to 715.62
WOMAC	*p*	0.001^*∗*^	0.049^*∗*^	0.001^*∗*^
	95% CI	7.26 to 15.30	0.01 to 8.13	11.60 to 18.90
Pain	*p*	0.001^*∗*^	0.515	0.05^*∗*^
	95% CI	2.06 to 4.30	−3.08 to 1.58	−0.06 to 4.92
Stiffness	*p*	0.001^*∗*^	0.769	0.001^*∗*^
	95% CI	0.72 to 2.64	−8.5 to 0.63	0.71 to 2.43
Physical function	*p*	0.003^*∗*^	0.024^*∗*^	0.001^*∗*^
	95% CI	2.25 to 9.61	0.62 to 8.31	7.44 to 13.35
CG				
OP	*p*	0.744	0.674	0.839
Perimeter (mm)	95% CI	−124.00 to 89.64	−133.29 to 202.86	−159.05 to 194.26
OP	*p*	0.068	0.033^*∗*^	0.012^*∗*^
Ellipse area (mm^2^)	95% CI	−6.72 to 178.15	12.72 to 280.85	55.75 to 409.25
CP	*p*	0.992	0.037^*∗*^	0.020^*∗*^
Perimeter (mm)	95% CI	−101.26 to 102.26	5.73 to 174.27	15.609 to 165.39
CP	*p*	0.357	0.001^*∗*^	0.014^*∗*^
Ellipse area (mm^2^)	95% CI	−143.86 to 386.14	206.08 to 591.07	70.88 to 581.84
WOMAC	*p*	0.935	0.944	0.975
	95% CI	−4.64 to 4.28	−3.01 to 3.22	−4.67 to 4.53
Pain	*p*	0.515	0.005^*∗*^	0.124
	95% CI	−2.41 to 4.70	0.52 to 2.62	−0.79 to 6.22
Stiffness	*p*	0.001^*∗*^	0.372	0.001^*∗*^
	95% CI	−3.47 to −0.61	−0.63 to 1.63	−2.84 to −1.23
Physical function	*p*	0.159	0.825	0.235
	95% CI	−1.07 to 6.21	−3.30 to 2.63	−1.55 to 6.05

^*∗*^Significant effect (*p* < 0.005). OP, open-eyes; CP, closed-eyes; BD, Baduanjin group; CG, control group; WOMAC, the Western Ontario and Mcmaster Universities Osteoarthritis Index; week 0, pretest; week 8, 8^th^ postassessment; week 12, 12^th^ postassessment.

## Data Availability

The datasets used and/or analyzed during the current study are available from the corresponding author upon reasonable request.
